# Circulating Galectin-1 and 90K/Mac-2BP Correlated with the Tumor Stages of Patients with Colorectal Cancer

**DOI:** 10.1155/2015/306964

**Published:** 2015-09-13

**Authors:** Keng-Liang Wu, Hong-Hwa Chen, Chen-Tzi Pen, Wen-Ling Yeh, Eng-Yen Huang, Chang-Chun Hsiao, Kuender D. Yang

**Affiliations:** ^1^Division of Hepatogastroenterology, Department of Internal Medicine, Kaohsiung Chang Gung Memorial Hospital, Kaohsiung 83301, Taiwan; ^2^Graduate Institute of Clinical Medical Sciences, Chang Gung University, Taoyuan 33302, Taiwan; ^3^Division of Colonic and Rectal Surgery, Department of Surgery, Kaohsiung Chang Gung Memorial Hospital, Kaohsiung 83301, Taiwan; ^4^College of Medicine, Chang Gung University, Taoyuan 33302, Taiwan; ^5^Department of Medical Research, Kaohsiung Chang Gung Memorial Hospital, Kaohsiung 83301, Taiwan; ^6^Department of Radiation Oncology, Kaohsiung Chang Gung Memorial Hospital, Kaohsiung 83301, Taiwan; ^7^School of Traditional Chinese Medicine, Chang Gung University College of Medicine, Taoyuan 33302, Taiwan; ^8^Institute of Clinical Medicine, National Yang Ming University, Taipei 11221, Taiwan; ^9^Department of Medical Research and Development, Chang Bing Show Chwan Memorial Hospital, Changhua 50544, Taiwan

## Abstract

*Background*. The simultaneous correlation of serum galectin-1, galectin-3, and 90K/Mac-2BP levels with clinical stages of patients with colorectal cancer has not yet been clarified. We plan to measure the serum levels of galectin-1, galectin-3, and 90K/Mac-2BP of patients at different stages of colorectal cancer and analyze the correlation of these galectins with stages of colorectal cancers. *Methods*. 198 colorectal cancer patients (62 ± 13 (range 31–85) years old, 43.6% female) were recruited for this study. Subjects' blood samples were checked for serum galectin-1, galectin-3, 90K/Mac-2BP, and carcinoembryonic antigen by sandwich enzyme-linked immunosorbent assay. We determined the correlation between plasma concentrations with clinical tumor stages. *Results*. Colorectal cancer patients with larger cancer sizes (stages T3, T4 rather than T1, T2) have higher serum 90K/Mac-2BP (*P* = 0.014) and patients with lymph node metastasis have higher serum galectin-1 (*P* = 0.002) but there was not a significant correlation between galectin-3 and tumor staging of colon cancer. In colorectal cancer patients even with normal carcinoembryonic antigen, serum galectin-1 could predict more lymph node metastasis. *Conclusions*. We found 90K/Mac-2BP correlated with the size of colorectal cancer. Galectin-1 but not galectin-3 was associated with lymph node metastasis. Galectin-1 could predict more lymph node metastasis in colorectal cancer patients with normal serum carcinoembryonic antigen.

## 1. Introduction

Carcinoembryonic antigen (CEA) was one of the first serological tumor markers to be discovered and has contributed significantly to the prediction of colorectal cancer and its recurrence. Several other serum markers in addition to CEA, such as carbohydrate antigen 19-9 (CA19-9), carbohydrate antigen 242, and tissue inhibitor of metalloproteinases type 1, have been reported and evaluated for various clinical uses in colorectal cancer (CRC) management. These serum markers may contribute to the prediction of prognosis or relapse after therapy, but high-powered, controlled studies are still needed to assess potential biomarkers for prognosis and recurrence of CRC. Serum markers for CRC are preferred over tissue- or stool-based assays, especially for screening and monitoring purposes, which require repeat testing. Blood-based tests have a better acceptance level and provide increased patient compliance.

Galectins are *β*-galactoside-binding proteins possibly involved in tumor prognosis. Four galectins, galectin-1, galectin-3, galectin-4, and galectin-8, are expressed in the human colon and rectum and their expressions show significant changes during colorectal cancer development and metastasis [1]. In particular, galectin-1 and galectin-3 play a role in the regulation of cell migration. Galectin-1, galectin-3, and their binding protein 90K/Mac-2BP are reported to be correlated and modulated with the malignancy prognosis and distal metastasis of colon cancer [2–4], but clinically the correlation between these serum galectins and prognosis of patients with colorectal cancer is less studied, especially 90K/Mac-2BP. Galectin-3 is also known to maintain epidermal growth factor receptor lattice formation on colon cancer cells for enhancing growth and epithelial mesenchymal transition that is implicated in cancer stem cells formation, resulting in the progression, and metastasis [5]. 90K/Mac-2BP, a tumor-associated glycoprotein, interacts with galectins and has roles in host defense by augmenting the immune response, but serum 90K/Mac-2BP level was suggested to indicate poor prognosis in several cancers [6–9]. There are few studies regarding serum 90K/Mac-2BP concentration on colon cancer prognosis reported to date. The aim of our study was to identify whether galectin-1, galectin-3, and/or 90K/Mac-2BP correlates with tumor staging in patients with CRC and to investigate their possible clinical role in the prediction of patients with CRC.

## 2. Methods

### 2.1. Subjects

One hundred ninety-eight patients with colorectal cancer who received surgical resection were enrolled in this study. Exclusion criteria included no well-defined pathology and no adequate clinical document available. The study was conducted at the Kaohsiung Chang Gung Memorial Hospital between May 2008 and June 2011, after the study protocol was approved by the Institutional Review Board of the hospital. All patients received surgical resection for CRC. Clinical data about age, gender, size of cancer, lymph node metastasis, and pathologic reports (vascular invasion, lymph node invasion, and well/poor differentiation) and clinical stage by TNM (tumor, nodes, and metastasis) system of the American Joint Committee on Cancer (AJCC 7th edition) were recorded as shown in Table 1. Blood samples were taken before the surgery and stored at −80°C until ELISA.

### 2.2. Serum Samples Studied

Serum samples were from the same patients who underwent colon cancer resection. These serum samples studied were obtained from the tissue bank of the Kaohsiung Chang Gung Memorial Hospital. One hundred ninety-eight consecutively decoded CRC patients (105 males and 93 females, 62 ± 13 years) who received surgery during May 2008 to June 2011 were enrolled in this study. Of the 198 patients, 174 had no clinically detectable metastasis (94 males and 80 females) and 24 had liver metastasis (11 males and 13 females). Serum galectin-1 and galectin-3 levels were assessed by a microplate immunoenzymatic method (Bender MedSystems, Vienna, Austria), and concentration of 90K/Mac-2BP molecule was evaluated using a commercial enzyme-linked immunosorbent assay kit (DIESSE, Siena, Italy) according to the manufacturer instructions.

### 2.3. Determination of Serum Galectins and 90K/Mac-2BP Concentrations

The 96-well plates were coated with anti-galectin antibody at 2.5 mg/mL in coating buffer (15 mmol/L Na_2_CO_3_ and 17 mmol/L NaHCO_3_, pH: 9.6) overnight at 4°C. The plates were washed with a washing buffer (0.05% Tween-20 in PBS) and incubated with blocking buffer (1% bovine serum albumin in PBS) for 1 hour at room temperature. Serum samples or standard recombinant galectins were introduced to the plates for 2 hours before the application of biotinylated anti-galectin antibody (1.25 mg/mL in blocking buffer) for 1 hour at room temperature. After introduction of ExtrAvidin peroxidase (1 : 10,000 dilution in blocking buffer) for 1 hour, the plates were developed with Sigma FAST OPD for 10 minutes. The reaction was stopped by adding 4 mol/L sulfuric acid, and the absorbance was read at 492 nm by a microplate reader. We measured the exact concentration of galectin-1 (R&D), galectin-3 (R&D), and 90K/Mac-2BP (Bender MedSystems) by intrapolation of a standard curve made by a series of well-known concentrations as per manufacturer's instruction.

### 2.4. Statistical Analysis

Data were reported as means ± standard errors of the mean unless otherwise indicated. Continuous and categorical variables were compared by using Student's *t*-test and *χ* ^2^ or Fisher's exact test, respectively. Spearman's rank correlation coefficient was used to explore relationships among variables. Predictability for an inadequate outcome when additional risk factors (*P* < 0.05 on multivariate logistic regression) were individually added was further determined.

## 3. Results

### 3.1. Serum Concentrations of Galectin-1, Galectin-3, and 90K/Mac-2BP

The 198 consecutive patients were male-predominant (105/198). Of the patients studied, more than one-half of the patients (100/198, 51%) had rectal cancer. Approximately one-half (46%, 91/198) had advanced cancer TNM systems III and IV and 44% (87/198) had lymph node metastasis.

### 3.2. Serum Galectin-1 and CEA Levels Associated with TNM System

**Figure 1:**
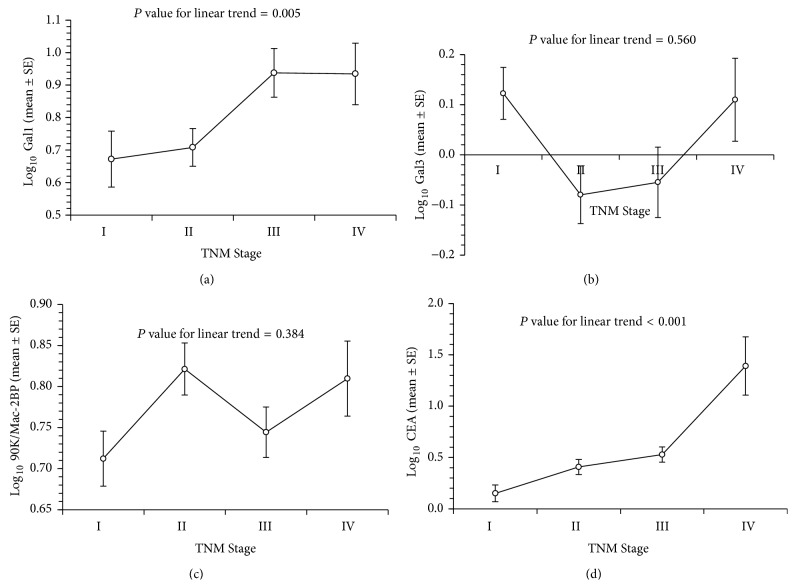
The correlation of galectin-1, galectin-3, 90K/Mac-2BP, and carcinoembryonic antigen with TNM system score. Galectin-1 levels were associated with advanced TNM Stages (III and IV, *P* = 0.005). Carcinoembryonic antigen levels were significantly associated with advanced stage of TNM system (*P* < 0.001). (d) Gal1: galectin-1; Gal3: galectin-3; Gal3BP: 90K/Mac-2BP; CEA: carcinoembryonic antigen.

Serum galectin-1 levels in CRC patients ranged from 0.14 to 198.34 (average 14.19 ± 2.63) ng/mL. Patients with TNM Stage III or IV revealed significantly higher galectin-1 levels (20.93 ± 3.77 ng/mL versus 8.73 ± 1.08 ng/mL). Galectin-3 and 90K/Mac-2BP levels were not significantly different between patients with lower (I or II) and advanced stages (Table 2). CEA levels had a higher trend in patients with advanced TNM Stage (26.3 ± 19.89 versus 302.39 ± 140.43 ng/mL). As shown in Figure 1, galectin-1 but not galectin-3 or 90K/Mac-2BP levels were significantly higher in CRC patients with TNM Stage III/IV. Higher galectin-1 levels were found in patients with Stage III/IV rather than Stage I and Stage II (*P* = 0.005) (Figure 1(a)). In addition, we also found that CEA concentrations were significantly correlated with TNM Stage of colon cancer (*P* < 0.0001) (Figure 1(d)).

### 3.3. 90K/Mac-2BP Levels Associated with Tumor Size (T Stage)

**Figure 2:**
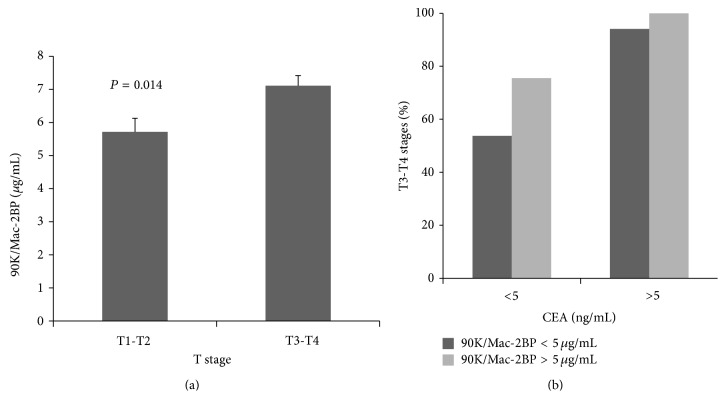
Patients with larger cancer sizes of stages T3, T4 rather than T1, T2 have more serum 90K/Mac-2BP levels which were significantly associated with tumor size (T3 or T4) (*P* = 0.014). Galectin-3 or galectin-1 levels were not significantly associated with tumor size. In colon cancer patients with normal serum carcinoembryonic antigen, advanced T stage had higher serum 90K/Mac-2BP levels. (b) CEA: carcinoembryonic antigen.

**Figure 3:**
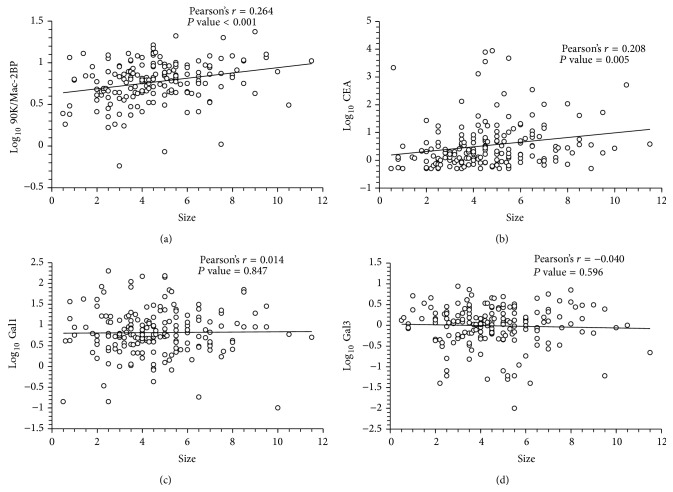
Linear correlation between tumor size of colon tumors and 90K/Mac-2BP levels (*r* = 0.264, *P* < 0.001) or carcinoembryonic antigen levels (*r* = 0.208, *P* = 0.005) but no significant correlation with galectin-1 or galectin-3 levels. Gal1: galectin-1; Gal3: galectin-3; Gal3BP: 90K/Mac-2BP; CEA: carcinoembryonic antigen.

The patients with advanced T stage (stages T3-T4) had higher serum 90K/Mac-2BP levels than those with small tumor size (stages T1-T2) (*P* = 0.014) as shown in Figure 2(a). In contrast, galectin-3 and galectin-1 levels were not significantly associated with tumor size (data not shown). Interestingly, we found that CRC patients with normal serum CEA and higher serum 90K/Mac-2BP levels were associated with tumor size (T3 or T4) (Figure 2(b)). Analyses of linear correlation between galectins levels and tumor size also found that tumor size was significantly correlated with 90K/Mac-2BP concentrations (*r* = 0.264, *P* < 0.001) (Figure 3(a)) and with CEA levels (*r* = 0.208, *P* = 0.005) (Figure 3(b)), but no significant correlation was found with galectin-1 or galectin-3 levels (Figures 3(c) and 3(d)).

### 3.4. Lymph Node Metastasis Associated with Galectin-1 and CEA Levels

**Figure 4:**
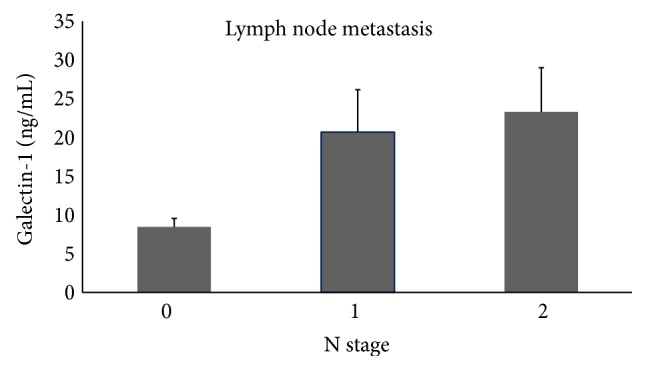
Serum galectin-1 concentrations were significantly increased in patients with lymph node metastasis (*P* = 0.002).

**Figure 5:**
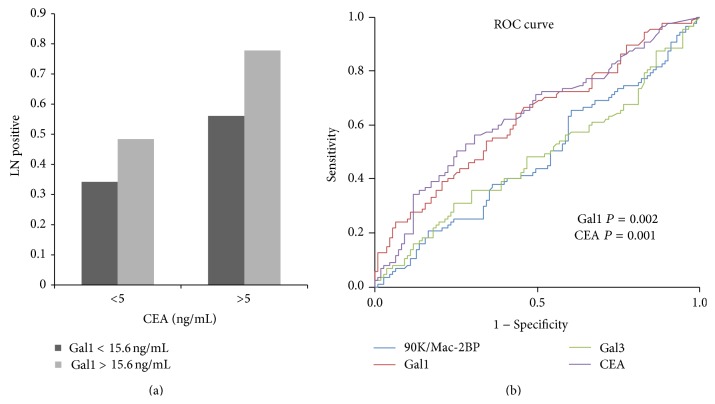
Galectin-1 levels predicted positive lymph node metastasis of colon cancer. (a) Analysis of Receiver Operating Characteristic curves found that galectin-1 and carcinoembryonic antigen levels could predict good sensitivity and specificity, showing area under the curve (AUC) at 0.627 for galectin-1 and at 0.638 for carcinoembryonic antigen. (b) Gal1: galectin-1; Gal3: galectin-3; 90K: 90K/Mac-2BP; CEA: carcinoembryonic antigen; ROC: Receiver Operating Characteristic.

Patients with lymph node metastasis had higher serum galectin-1 levels (*P* = 0.002, Figure 4). However, galectin-3 or 90K/Mac-2BP levels were not correlated with lymph node metastasis (data not shown). Further analysis found that, in patients with normal CEA concentration, galectin-1 levels predicted positive lymph node metastasis of colon cancer (Figure 5(a)). Analysis of Receiver Operating Characteristic (ROC) curves of galectins and CEA concentrations for CRC with LN metastasis found that galectin-1 and CEA levels could predict lymph node metastasis at good sensitivity and specificity, showing area under the curve (AUC) at 0.627 for galectin-1 and at 0.638 for CEA (Figure 5(b)).

## 4. Discussion

Galectin-3 and galectin-1 are members of the galectins gene family that are expressed at elevation in a variety of neoplastic cell types and have been associated with cell growth, apoptosis, cellular adhesion process, cell proliferation, transformation, invasiveness, and metastasis. Some galectins (such as galectin-1 and galectin-3) and their binding proteins such as 90K/Mac-2BP are reported to be correlated with the malignancy prognosis and distant metastasis of colon cancer [2–4, 10–12]; Nakamura et al. had clarified that strong expression of galectin-3 in colorectal cancer correlated with cancer progression, liver metastasis, and poor prognosis [13]. Several reports have indicated its involvement in carcinogenesis of certain cancers [14, 15], but we did not find any correlation between serum galectin-3 levels and progression of colon cancer in our study. Thijssen et al. showed that galectin-3 expression on epithelium of colon cancer was unaltered [16] and Sanjuán et al. found that galectin-3 expression is downregulated in the initial stages of neoplastic progression, whereas a dissociated cytoplasmic expression increases in later phases of tumor progression [3]. Galectin-3 was a cytosolic and secretary protein but was less detected in the extracellular medium during primary cancer cell culture [17], indicating that cancer cells alone do not secrete galectin-3 into extracellular medium and this could be the reason why the present result, serum galectin-3, was not correlated with prognosis of colon cancer. Further studies to clarify are necessary.

We found that 90K/Mac-2BP could be a predictor for the advanced invasion of colorectal cancer. Greco et al. showed that galectin-3 ligand, 90K/Mac-2BP molecule, was increased in the blood plasma from patients with both adenomatous and adenocarcinomatous lesions [18]. Iacovazzi et al. also clarified the correlation prognosis of colon cancer with 90K/Mac-2BP [6], the same result as our present study, but we did not find that 90K/Mac-2BP mean values ranged higher in right-side than in left-side colon cancer although they hypothesized that this could be due to the better blood and lymph supply that can provide a more efficient local tumor defense. Natoli et al. showed that the increase of 90K/Mac-2BP after r-IFN-alpha-2b administration might be of importance for the early detection of disease recurrence in breast and colon cancer patients without evidence of disease [19]. In contrast, Lee et al. clarified that 90K/Mac-2BP itself has antitumor activity in CRC cells via suppression of Wnt signaling with a novel mechanism of ISGylation-dependent ubiquitination of beta-catenin when it interacts with CD9/CD82 but is downregulated in advanced CRC tissues [20]. To date, the effect of 90K/Mac-2BP on colon cancer prognosis is not well studied.

In this study, we found that galectin-1 could be a marker for prediction of lymph node metastasis. Barrow et al. showed that the concentrations of galectin-1 were not significantly increased in patients with colorectal cancer [12] but Thijssen et al. showed that endothelial cells express galectin-1 and that the expression and distribution change on cell activation, resulting in a different profile in the tumor vasculature [16]. The expression of galectin-1 significantly increased with the degree of dysplasia from Hittelet A study [21]. Nagy et al. observed a significant prognostic value associated with galectin-1 in Dukes A and B colon tumors [4] and our study showed different result. From André et al.'s study, histopathological analysis of lymph node carcinomas indicated a correlation of either increased galectin-1 binding or reduced binding of both galectins with the occurrence of lymph node lesions [22]. It could be that galectin-1 in colorectal mucosa is predominant in stromal cells whose overexpression is associated with the neoplastic progression of colorectal cancer as told by Sanjuán et al. [3]. We also found that, in colon cancer patients with normal CEA, galectin-1 could predict more lymph node metastasis.

In conclusion, serum 90K/Mac-2BP concentrations in colorectal cancer patients at an advanced stage were significantly higher than those at an early stage. Higher galectin-1 concentration was correlated with lymph node metastasis, especially in colorectal cancer patients with normal CEA. These results suggest that the measurement of serum 90K/Mac-2BP and galectin-1 concentration is helpful to predict patients with advanced colorectal cancer.

## Figures and Tables

**Table 1:** Baseline characteristics of patients of the study.

	Number
Gender (male/female)	105/93
Age (mean [range])	62 ± 13 [31–85] years
Site (A/T/D/S/R)	33/15/12/38/100
Histopathology	5/176/17
(well/moderate/poor differentiation)	
Stage (TNM)	45/62/67/24
(I/II/III/IV)	
Vascular invasion	39/198 (19.7%)

Note: A: cecum and ascending; T: transverse colon; D: descending colon; S: sigmoid colon; R: rectum.

**Table 2:** Levels of galectin-1, galectin-3, 90K/Mac-2BP, and CEA in the sera of colon cancer patients.

	All patients	Stages I and II	Stages III and IV	*P *
Galectin-1 (ng/mL)	14.33 ± 1.67	8.73 ± 1.08	20.93 ± 3.77	0.002^*^
Galectin-3 (ng/mL)	1.54 ± 0.10	1.44 ± 0.11	1.65 ± 0.17	0.320
90K/Mac-2BP (*μ*g/mL)	6.77 ± 0.25	6.87 ± 0.36	6.65 ± 0.36	0.665
CEA (ng/mL)	153.19 ± 66.31	26.30 ± 19.89	302.39 ± 140.43	0.055

CEA: carcinoembryonic antigen; ^*^significant difference.

The continuous variables were described by mean ± SEM.
